# The surgical effect and safety of a novel intraocular choroidal melanoma resection

**DOI:** 10.3389/fmed.2025.1554581

**Published:** 2025-05-30

**Authors:** Yulun Ou, Ying Li, Xianfen Cao, Guoping Kuang

**Affiliations:** ^1^Department of Ophthalmology, The First People’s Hospital of Chenzhou, Chenzhou, Hunan, China; ^2^Department of Science and Education, The Affiliated Hospital of Xiangnan University, Chenzhou, Hunan, China; ^3^Aier Eye Hospital affiliated with Jinan University, Shenzhen, Guangdong, China

**Keywords:** choroidal melanoma, vitrectomy, resection, hyphema, metastasis

## Abstract

**Purpose:**

To investigate the surgical effect and safety of a novel technique for the excision of intraocular choroidal melanoma in order to reduce the risk of serious adverse events.

**Methods:**

This retrospective study analyzed 23 patients with choroidal melanoma (23 eyes) from January 2016 to December 2022. Instead of the standard peripheral retina incision and subsequent tumor removal, we performed phacoemulsification and complete vitrectomy with tumor and overlying retina removal under high intraocular pressure. Without further retinal reattachment, the basal sclera is preserved. The tumor was resected, leaving 1–2 mm of the surrounding normal retina and choroid. The resection edge was further treated with laser therapy, which was followed by the replacement of heavy water with silicone oil. Finally, the vitreous cavity was filled with silicone oil. The median operation time was 1.5 h (1.2–2.5 h). Complete ophthalmic examinations were performed 1 day, 1 week, and 1, 3, and 6 months postoperatively. Systemic examination was conducted every 6 months.

**Results:**

The median duration for all surgeries was 1.5 h (range: 1.2–2.5 h). Minor bleeding occurred at the mass resection margin intraoperatively, and the minimal-to-little hyphema observed on postoperative day 1 in all patients was absorbed 3–7 days later. No retinal detachment was noted at a mean follow-up of 42.5 ± 6.9 (range, 36–60) months. The best-corrected visual acuity at the last follow-up was lower than that before surgery (*P* = 0.001), One patient required enucleation due to intraocular recurrence, and one patient died from metastatic choroidal melanoma. The remaining patients remained healthy during the follow-up period.

**Conclusion:**

Maintaining a vitreous cavity filled with half air and half heavy water while excising the choroidal tumor and the overlying retina is a simple, effective, and safe surgical approach.

## Introduction

Uveal melanoma (UM) is the most common primary intraocular malignancy in adults, with a worldwide incidence rate of 0.38–6.0/million per year (1–6). Approximately 80% of these occur in the choroid. Enucleation is the most common intervention method for choroidal melanoma, particularly for larger UM and lesions that respond poorly to radiotherapy (7–9). However, the enucleation injury caused for small and medium choroidal tumors is large and seriously affects the appearance and visual function, especially for patients with cyclopia. Topical therapy is gaining increasing attention (10). Many local treatment methods, including Proton Beam Therapy (11), Transpupillary Thermotherapy (TTT) (12), photodynamic therapy (13), immunotherapy (6), target therapy (14), radiotherapy, local excision, and multi-treatment combinations, were used (15), aiming to preserve the eyeball and as much vision as possible in the premise of tumor control (Table 1). Patchy radiotherapy is as effective in preventing death from medium-sized choroidal melanoma as enucleation (16, 17), making it the most common first-line treatment for small and medium tumors (height ≤ 10 mm, base diameter ≤ 15 mm) (2, 18). Brachytherapy is usually performed using ruthenium (Ru-106) or iodine (I-125) that can be delivered as charged particles or stereotactic radiotherapy (2, 8). The 5 years local control rate can achieve 85%–95% for small and medium-sized tumors located in the middle and peripheral regions. However, in cases where the tumor is large, it becomes challenging to achieve complete radiotherapy due to the inadequate control of the radiotherapy dose. This often leads to residual tumor tissue, and it is also difficult to position the radiotherapy device in the posterior region. The primary complications associated with this treatment include radiation-induced injuries such as radiation retinopathy, optic neuropathy, neovascular glaucoma, and vitreous hemorrhage. Notably, the rate of long-term vision preservation (vision > 20/200) within 5 years is approximately 40%, which is considered superior to resection (19). Local resection offers a more comprehensive approach for treating localized tumors with distinct boundaries. It promptly eliminates lesions and boasts a high control rate, effectively removing the tumor or minimizing the chances of recurrence and metastasis. According to Shields (20) and other studies, the 5 years control rate following local surgical resection ranges from 70% to 85%. However, when tumor thickness exceeds 8 mm, the recurrence rate increases significantly. Consequently, some experts recommend intraocular resection as an alternative to brachytherapy and partial resection of the posterior segment for restrictive cases (8, 21, 22). Additionally, concerns have been raised about the possibility of intraoperative tumor cell dissemination during local resection, which could potentially lead to recurrence. Nevertheless, relevant studies have found no significant differences in metastasis rates or mortality after radiotherapy compared to local tumor resection (6, 9, 23). D’Amato et al. demonstrated that the 5 years metastasis rate following local resection was approximately 20%–25%, a figure that did not significantly diverge from that of radiotherapy (24). Similarly, Jampol et al. observed that the long-term metastasis rate after radiotherapy (roughly 30% over 10 years) was comparable to that of surgical intervention (25). The Collaborative Ovarian Melanoma Study (COMS) revealed that the risk of metastasis is primarily associated with tumor gene characteristics (such as chromosome three monomer) and the maximum basal diameter, rather than the specific treatment approach (26). Furthermore, the findings of Lazaros Konstantinidis’s research do not endorse the theory that intraocular choroidal tumor resection leads to the widespread dissemination of melanoma within the entire eye and system (27). Instead, it suggests that objections to surgical resection are rooted in a mechanistic understanding of metastatic spread.

**TABLE 1:** Comparative analysis of treatment methods for choroidal melanoma.

Treatment method	Plaque Brachytherapy	Proton Beam Therapy	Stereotactic radiosurgery (SRS)	Local resection	Laser Therapy	Cryotherapy	Photodynamic therapy (PDT)	Immunotherapy	Targeted therapy
Indications	Tumor height < 10 mm, base diameter < 16 mm.	Tumors suitable for precise proton beam irradiation.	Tumors suitable for high-precision radiation therapy.	Tumors with locations and sizes suitable for surgical resection.	Small tumors with height < 3 mm, no retinal detachment on the surface.	Locally larger but superficial tumors.	Small tumors with thickness < 4 mm, diameter < 10 mm.	Mainly for metastatic choroidal melanoma (mUM), especially HLA-A0201-positive patients.	Patients with specific genetic mutations (e.g., GNA11/GNAQ).
Complexity	High technical requirements; precise dose planning and placement are needed.	Requires high-precision equipment and technical support; high complexity.	High technical requirements; precise dose planning and positioning are needed.	High surgical complexity; tumor location and size must be considered.	Relatively simple operation, but precise localization is required.	Relatively simple operation, but precise localization is required.	Combined with specific drugs and light sources; moderate complexity.	Requires genetic testing support; complex treatment process with monitoring of immune-related adverse events.	Requires genetic testing support; complex treatment process with monitoring of targeted therapy-related adverse reactions.
Postoperative complications	Radiation retinopathy, radiation maculopathy, radiation optic neuropathy.	Rare, but may include retinopathy and optic nerve damage.	Optic nerve damage, retinopathy, and other radiation-induced injuries.	Hemorrhage, infection, retinal detachment.	May cause retinal damage or vision loss.	Cryotherapy-related retinal damage, retinal detachment.	May cause retinal edema, vision loss.	Immune-related adverse events (e.g., skin toxicity, endocrine toxicity).	May cause skin toxicity, gastrointestinal reactions, etc.
Tumor recurrence rate	High local control rate; low recurrence rate.	High local control rate; low recurrence rate.	High local control rate; low recurrence rate.	Low recurrence rate, but close follow-up is needed.	Suitable for small tumors; low recurrence rate.	Suitable for local tumors; low recurrence rate.	Suitable for small tumors; low recurrence rate.	Higher recurrence rate, but combination therapy may improve prognosis.	Recurrence rate depends on genetic mutation type and treatment regimen.
Visual acuity preservation rate	Visual acuity preservation rate depends on tumor location, size, and radiation dose.	Higher visual acuity preservation rate, especially for tumors near the optic nerve.	Higher visual acuity preservation rate, but strict control of radiation dose is required.	Higher visual acuity preservation rate, but close monitoring is required postoperatively.	Higher visual acuity preservation rate, especially for small tumors in the posterior pole.	Moderate visual acuity preservation rate, depending on tumor location and size.	Higher visual acuity preservation rate, especially for small tumors in the posterior pole.	Not applicable (mainly for metastatic patients).	Not applicable (mainly for metastatic patients).

Despite the advantages of intraocular resection, the surgical procedure for choroidal melanoma requires pars plana vitrectomy to remove the tumor, potentially causing postoperative vitreous or retinal hemorrhage, retinal detachment, persistent high intraocular pressure or cataract. Additionally, there exists a significant risk of vision loss or impairment following the resection of the tumor located in the posterior pole, whereas anterior tumors may exhibit better visual preservation.

In summary, plaque radiotherapy demonstrates greater stability in controlling small and medium-sized tumors located in the anterior region, whereas local resection is ideal for small and medium-sized tumors with distinct boundaries in the posterior position. No notable disparity in metastasis rates was observed between the two treatment groups, metastasis primarily depend on the tumor’s biologic behavior. Regarding complications, radiation injury predominantly occurs in radiotherapy, whereas the risks associated with local resection are primarily during the perioperative period. However, advancements in technology have reduced the duration and risks associated with minimally invasive surgeries. This study evaluated an intraocular surgical technique for excising the choroidal tumor and the overlying retina with the vitreous cavity maintained as half gas and half heavy water, aimed at minimizing surgical complexity and postoperative complications.

## Materials and methods

### Study design

This is a retrospective study. We analyzed 23 patients (23 eyes) diagnosed with choroidal melanoma from January 2016 to December 2022 at the Department of Ophthalmology, The First People’s Hospital of Chenzhou, All procedures adhered to the tenet of the Declaration of Helsinki and obtained approval from the hospital’s ethical committee (No: CYKY202112010). Written informed consent was provided by each patient before study.

### Participants

**FIGURE 1:**
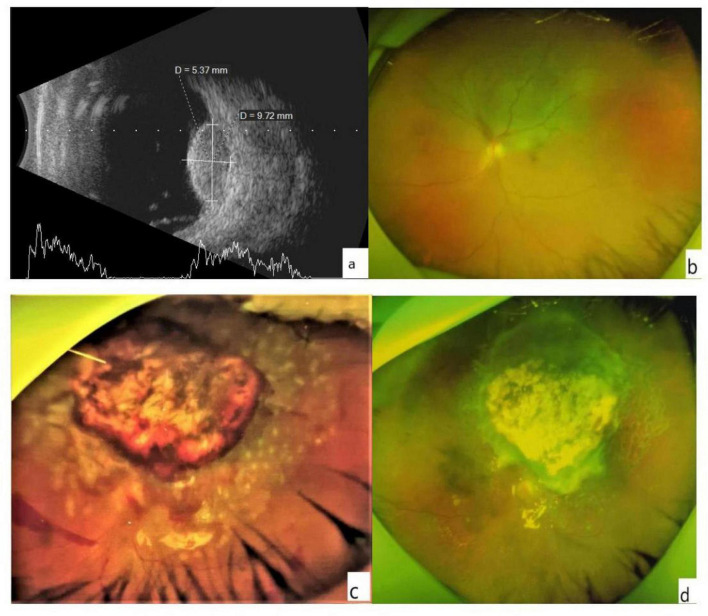
**(a)** The B-scan image shows the height and basis dimensions of the tumor. **(b)** An ultra-wide-angle photo of the fundus. The choroidal mass in the superior retina appears as a gray bulge with some yellowish-white pigmentation on the surface, and retinal detachment close to be macula and optic dick can be seen. **(c)** Minor hemorrhage was visible at the edge of the retinochoroidectomy area on postoperative day 1. The mass resected retinal margin was closed with a laser. **(d)** A month later, no apparent of the tumor was noted in the resected area, and no retinal detachment was detected.

Data available for all patients included patient characteristics, systemic diseases, and ophthalmic history. Ocular examination performed at baseline and at each follow-up visit (1 day, 1 week, 1, 3, and 6 months after surgery, and every 6 months thereafter) included BCVA, careful slit-lamp examination, Goldmann applanation tonometry, fundus photography, and A- and B-scan ultrasonography. Orbital magnetic resonance imaging (MRI) and metastatic screening that included serum biochemical analysis, liver function examination, abdominal ultrasonography, abdominal computed tomography (CT), and chest radiography were done every 6 months. The measurements were collected at the time the examination was performed. All ultrasonographic recordings were reassessed for the maximal tumor height and basal diameter (dimensions) before inclusion in this study (Figures 1a, b). The diagnosis of UM was confirmed in all cases postoperatively by pathological analysis. The exclusion criteria for intraocular resection included extraocular or distant tumor metastasis, tumors exceeding the equator or involving the serrata margin for up to two-thirds of the ciliary bodies, tumor diameter greater than 10 mm, systemic conditions that could not tolerate surgery, and diffuse melanoma. The same surgeon performed all intraocular surgeries.

### Intervention

The procedure started with standard phacoemulsification and posterior chamber intraocular lens implantation. A 23-G or 25-G pars plana vitrectomy (CONSTELLATION, Alcon Vision LLC, Fort Worth, TX, United States) was performed. We removed the posterior and base vitreous completely by staining the cortical vitreous with diluted triamcinolone. Retinal vascular electrocoagulation was performed approximately 1 mm into the normal tissue surrounding the tumor, the overlying retina was cut, revealing the choroidal tumor. Electrocoagulation was applied to the normal choroid 1 mm around the tumor. Heavy water (DK-Line, Bausch & Lomb Inc., Königsbrunn, Germany) was injected to fully cover the tumor area up to anterior to the equator. Air-fluid exchange was then performed to aspirate part of the infusion, filling the anterior vitreous cavity with air. Excision of the tumor began at its center and proceeded outward. If bleeding occurred, the air perfusion pressure was increased to 60 mmHg, and paused for 2–3 min. Hemorrhagic vitreous fluid was aspirated, and heavy water should be replenished as needed to maintain coverage of the tumor as it was excised down to the white scleral tissue. The vitreoretinal cutter was then used for circumferential polishing of the scleral surface to remove residual pigmented tissue, macroscopically suspicious tumor tissue, and 1 mm of surrounding normal choroidal tissue. Currently, there is no clear standard on how much normal tissue around a tumor should be removed to reduce the likelihood of recurrence. So, we referred to the resection range for intraocular surgery of choroidal melanoma as reported in previous literature for the resection (21). Continuous intraocular laser photocoagulation (500 mW) was performed on the scleral bed and the excision margins were treated with 3–4 rows of laser photocoagulation (150–200 mW). After heavy water-silicone oil exchange, the vitreous cavity was filled with silicone oil (DK-Line, Bausch & Lomb Inc., Königsbrunn, Germany). The puncture port was sutured using 8-0 absorbable sutures (polyglactin 910; Ethicon Inc, Johnson & Johnson, Somerville, NJ, United States). The material collected during the procedure was centrifuged, and cell blocks were prepared, formalin-fixed, paraffin-embedded, and submitted for pathology studies.

Postoperatively, all patients underwent BCVA assessment, slit-lamp microscopy, indirect ophthalmoscopy, and ultra-wide angle fundus photography. Follow-up examinations were performed 1 day, 1 week, and 1, 3, and 6 months after the surgery, and every 6 months thereafter. Ultrasonographic biomicroscopy (UBM) and B-scan ultrasonography were performed every 6 months for at least 2 years to evaluate the eyes for tumor regrowth and dissemination. Systemic blood tests and liver imaging were also performed. The silicone oil was removed about 1–3 months after the operation when the eye condition was stable, the retina was flat, and there was no apparent recurrence of the tumor.

### Statistical analyses

Descriptive statistical results are presented as mean ± standard deviation (SD). Quantitative variables were compared by the paired-samples *t*-test. The BCVA was expressed as the standard logarithmic value of VA, converted to the logarithm of the minimum angle of resolution (logMAR) for statistical analysis. A *p*-value < 0.05 was considered statistically significant. Statistical analysis was performed using GraphPad Prism 9.0.

## Results

Detailed basic characteristics of the 23 patients before surgery are shown in Table 2.

**TABLE 2:** Basic characteristics of 23 patients with choroidal melanoma.

Characteristics	Results [*n* = 23 (%)]
Age, year (mean ± SD)	61.87 ± 6.18
**Sex [*n* (%)]**	
Male	12 (52.2)
Female	11 (47.8)
**Eye [*n* (%)]**	
Right	7 (58.33)
Left	5 (41.66)
**Tumor (mean ± SD)**	
Height, mm	3.95 ± 1.02
GBD, mm	6.20 ± 1.82
Distance to optic, mm	4.15 ± 0.68
**Visual acuity**	***n* (%)**
≥ 100/200	1 (4.35)
< 60/200–100/200	4 (17.40)
< 20/200–60/200	6 (26.08)
< 20/200	12 (52.17)

GBD, greatest basal dimension; SD, standard deviation; BCVA, best-corrected visual acuity.

The 23 affected eyes were followed up for 42.5 ± 6.9 (range, 36–60) months. The postoperative BCVA (logMAR) was 0.96 ± 0.63, significantly different from the preoperative BCVA. The preoperative and postoperative IOP values were similar (Table 3). Only a small amount of bleeding from the resection margins was noted postoperatively in all patients (Figure 1c), these were self-absorbed within 2–4 weeks (Figure 1d). A total of 18 patients had decreased visual acuity, three patients had no significant change, and two patients had improved visual acuity. One patient experienced local recurrence 30 months postoperatively. The patient with recurrent tumor underwent enucleation as requested, and histopathological examination after enucleation confirmed the recurrence. One patient died 3 years after surgery due to metastatic melanoma. The eye-retention rate in this study was 95.6%.

**TABLE 3:** IOP and BCVA at the last follow-up visit compared to those before surgery (*n* = 23).

Intraocular pressure/Visual acuity	Before surgery	At the last postoperative	t	*P*
IOP	19.55 ± 3.99	18.30 ± 1.92	1.68	0.107
BCVA (logMAR)	0.96 ± 0.63	1.42 ± 0.98	−3.93	0.001

IOP, intraocular pressure; BCVA, best-corrected visual acuity; SD, standard deviation; LogMAR, logarithm of the minimum angle of resolution.

## Discussion

Intraocular resection is relatively novel among the numerous choroidal melanoma treatments, allowing eye and residual visual function preservation following tumor resection without significant long-term complications (28–30). There is no report that intraocular resection has a higher tumor recurrence rate than brachytherapy (31), while it presents more benefits than other treatment modalities such as enucleation and brachytherapy (32, 33), including improved patient quality of life. As the understanding of tumor management improves (e.g., local resection with little impact on survival) (34, 35), intraocular surgical resection was gradually adopted, accepted, and proved effective by numerous studies, suggesting it should be considered even as the first choice for choroidal melanoma (10, 36–38). However, the possibility that intraocular resection would lead to tumor cell dissemination and recurrence or increased systemic disease remains controversial (39). In this study, the 23 patients who met the inclusion criteria, underwent intraocular resection as the first-line treatment found one local recurrence during the follow-up period (42.5 ± 6.9 months), similar to the results of Karkhaneh et al. (8). However, the efficacy in our study was better than the findings of Damato et al. (37), who performed intraocular resection on 61 choroidal melanomas and reported 12 tumor recurrences after surgery, a recurrence rate of 19%.

The literature reports that choroidal melanoma melanocytes invade the vitreous body and begin to shed pigment fragments into the vitreous during surgery (31, 40, 41). However, the pathological analysis did not find this phenomenon in any of the samples in this series. We performed lens removal to achieve better base vitrectomy, reduce the vitreous residue at the base, and avoid tumor cell implantation. Our approach involves maintaining the upper portion of the vitreous cavity as gas while the lower section remains filled with heavy water. Tumor resection is then carried out within this environment of air pressure and heavy water. It is essential to keep the tumor resection site continuously bathed in heavy water, as it promotes hemostasis more effectively than conditions of high hydrostatic pressure. This is because heavy water prevents blood from infiltrating the vitreous cavity, thereby minimizing any potential interference with surgical visibility. The presence of air serves to hinder the spread of tumor cells. During the resection procedure, tumor debris particles remain stationary, unaffected by the airflow within the vitreous cavity, and do not spill out through the puncture port due to the perfusion fluid outside the eye. Additionally, the density of the underwater environment prevents retinal detachment from occurring and expedites the surgical process. Furthermore, it obstructs gas from penetrating the venous circulation, which could potentially lead to fatal gas embolism by tearing the vortex vein under elevated pressure. This phenomenon has been documented as venous air embolism (42, 43) and perfluorocarbon syndrome (44). Retinal removal with a small margin around the tumor surface under high pressure may have reduced remnant pigment tumors localized on the outer retinal surface and the likelihood of recurrence. It is well-known that the nutrition of the outer five layers of the retina comes from the choroid. Even if one preserves the retina within the mass limits, it does not retain its value postoperatively because it lacks nourishment from the corresponding choroid, leading to outer retinal cell necrosis and loss of retinal function. Although a peripheral 180° retinotomy was performed with retinal resetting after removing the tumor, the retina might detach again after removing the silicone oil because of the anterior proliferative vitreoretinopathy (PVR) and other reasons (45). Laser closure was performed around the tumor resection area, resulting in no retinal detachment. Our surgical approach was relatively simple and maybe less time-consuming than other methods for preserving retinal tissue in the tumor area. We observed no intrascleral invasion postoperatively, possibly due to our careful handling of the tumor, removal of potentially invaded tissue from the periphery, and use of high-energy laser photocoagulation in the scleral bed.

Subretinal effusion at the periphery of the tumor leading to localized exudative retinal detachment was present in four of the patients before treatment; however, none developed retinal detachment after surgery, probably due to our more complete vitrectomy, careful management of the retinal margins after tumor resection, and complete tumor removal. Two cases had postoperative retinal or choroidal hemorrhages in the tumor resection margins, but these were absorbed approximately 2–4 weeks after surgery, possibly due to a reduction in postoperative IOP compared to the intraoperative IOP. Although three patients had high IOP preoperatively, only one had high IOP postoperatively, which normalized with aggressive anti-inflammatory and anti-glaucoma drug treatment.

Our patients had poor final visual acuity, probably due to the size and location of the tumors, which were mostly relatively close to the macula. Patients with large or posterior pole tumors, especially those in or affecting the macula, have a poor visual prognosis. Previous studies have identified postoperative rhegmatogenous retinal detachment as a significant factor influencing postoperative visual acuity. However, in this study, the utilization of triamcinolone acetonide suspension marked the vitreous and facilitated its complete removal. This approach resulted in minimal intraoperative bleeding, a clear visual field, absence of iatrogenic retinal holes beyond the tumor resection area, and importantly, no postoperative retinal detachment. Additionally, it mitigated the incidence of proliferative vitreoretinopathy (PVR). Some patients experienced further vision decline due to tumors near the macula. Many patients were diagnosed with fundus tumors after reporting vision loss. After tumor excision, excessive scar formation at the excision edges may lead to foveal depression traction toward the scar edge or laser spot expansion, or epiretinal membrane formation due to inflammation, causing further vision decline. Compared to large cohorts of patients treated with radiotherapy (46), most patients with small and medium-sized tumors maintained better vision than 20/200 at 36 months post-treatment. Vision after radiotherapy was slightly better than after intraocular excision, possibly because these patients did not have long-term radiation-related complications (over 3 years) (47).

During the excision process, we consistently maintained the wound surface below heavy water to isolate the gas from the wound. This prevented the possibility of compressed gas entering the circulatory system through torn vortex veins or abnormal choroidal venous openings during tumor removal, which could lead to ophthalmic venous air embolism (OVAE) and even severe systemic complications that threaten life. However, in this study, no patients experienced complications related to high pressure, particularly vascular air embolism issues (48, 49).

In this study, one patient experienced local recurrence 30 months postoperatively. The patient with recurrent tumor underwent enucleation as requested, and histopathological examination after enucleation confirmed the recurrence. One patient died 3 years after surgery due to metastatic melanoma. We consider that metastasis might have been present preoperatively but went undetected, rather than being induced by the surgery. Compared to the surgical method in Hamza H’s study (50), our surgery is simpler and needs less complex surgical skills. However, our study has many limitations. The patient selection criteria were strict, the choroidal tumors were relatively small, the sample size was small, the post - operative follow - up was short, and the results were from a single center. So, the low recurrence and mortality rates we observed might be biased. Also, the visual prognosis in our study was lower than that in other literature, probably because most tumors were located in the posterior pole. Only further multi - center, large - sample, and long - term studies can tell if this surgical technique significantly affects survival rates.

## Conclusion

This novel technique for choroidal tumor excision reduces intraoperative bleeding, prevents tumor cell dissemination, and avoids severe adverse events like ophthalmic venous air embolism. However, due to this study’s limitations, the technology’s impact on tumor recurrence and survival rates can only be assessed by future multi - center, large - sample, long - term follow - up studies.

## Data Availability

The raw data supporting the conclusions of this article will be made available by the authors, without undue reservation.
